# Rasburicase vs. allopurinol: mortality in hematological malignancies post anti-hyperuricemic therapy - real-world study

**DOI:** 10.1007/s00520-025-10090-y

**Published:** 2025-11-13

**Authors:** Mitchell Stuart Cairo, Jack Ray Gallagher, Yvonne Barnes, Edward Drea, Christopher Clark, Susan Carroll

**Affiliations:** 1 Departments of Pediatrics, Medicine, Pathology, Microbiology and Immunology and Cell Biology and Anatomy, Maria Fareri Children’s Hospital Westchester Medical Center (WMC), New York Medical College, 40 Sunshine Cottage Road, Valhalla, 10595 NY USA; 2 Clarity Pharma Research LLC, 511 Audubon, Spartanburg, SC 29302 USA; 3 Sanofi, Cambridge, 450 Water Street, MA 02141 USA

**Keywords:** Liquid tumor, Hematological malignancy, Tumor lysis syndrome (TLS), Hyperuricemia (HUA), Propensity score (PS), Retrospective, Real-world (RW)

## Abstract

**Purpose:**

This retrospective, real-world (RW) study utilizing propensity score matching investigated tumor lysis syndrome (TLS)-related mortalities following anti-hyperuricemic (HU) monotherapy (allopurinol vs. rasburicase) in hematological malignancies. The study aim was to determine if a significant difference exists in the proportion of TLS-associated mortalities in hematological malignancy patients who received either rasburicase or allopurinol.

**Methods:**

Following random selection of patient cases of rasburicase or allopurinol monotherapy, 141 in each group were PS-matched for TLS risk using 11 predictive covariates.

**Results:**

Rasburicase-treated patients had significantly lower TLS-associated mortality (2.1% (*n* = 3) vs. 7.1% (*n* = 10) (*p* = 0.047)).

**Conclusions:**

Following successful PS matching of TLS-risk factors, this study suggests rasburicase statistically significantly reduces TLS-associated mortalities compared to allopurinol.

## Introduction

Tumor lysis syndrome (TLS) is one of the common oncological emergencies encountered and is a potentially life-threatening condition that occurs in adults and children [1–3]. TLS may occur spontaneously with a heavy tumor burden or following initiation of cytotoxic therapy and rapid destruction of malignant cells with abrupt release of potassium, phosphorous, and nucleic acids that are metabolized into hypoxanthine, xanthine, and uric acid (UA). Hyperkalemia can cause dysrhythmias; hyperphosphatemia can cause hypocalcemia, leading to tetany, dysrhythmia, and seizure, and precipitation of calcium phosphate in various organs. UA can induce acute kidney injury by intrarenal crystals and by crystal-independent mechanisms such as renal vasoconstriction [4–7].

The incidence of TLS appears to be increasing [8–13], and TLS mortality is understudied. Out of a large national inpatient database, Pathak et al. reviewed the National Inpatient Sample Database from 2009 to 2011 in patients ≥18 years of age with a diagnosis of TLS. Pathak et al. integrated the National Inpatient Sample Database from 2009 to 2011 and identified 997 inpatient admissions with the diagnosis of TLS and demonstrated an associated inpatient mortality rate of 14.4% [14]. Moreover, a more recent paper (2019) has noted that the incidence of TLS is not well defined because of a historical lack of standardized diagnostic criteria and the variability of patient populations and treatment regimens. Monoclonal antibodies (mAbs) and tyrosine kinase inhibitors (TKIs) are two examples of widely used targeted therapies that have been shown to induce TLS as single-agent therapies or in combination with conventional chemotherapies. Morbidity and mortality may be higher with newer therapies because of a lack of recognition, inadequate prophylaxis, and delayed treatment [15]. The most widely used TLS diagnosis criteria were developed by Cairo and Bishop and account for the severity of illness from grade 0 (asymptomatic) to grade 5 (death) [16]. TLS risk evaluation and prophylaxis have been further addressed by the recommendations developed through an international panel of TLS experts convened by Cairo and others [17, 18].

Rasburicase and allopurinol are commonly used for the prevention or treatment (Tx) of TLS, but comparisons of these therapies are limited [19, 20]. In a retrospective, observational study of 26 rasburicase-treated hospitalized pediatric and adult patients with clinical or laboratory TLS who were propensity score (PS) matched with 104 allopurinol-treated patients, Cairo et al. found that rasburicase was significantly more effective than allopurinol in reducing uric plasma levels and in treating hyperuricemia (HUA) [21]. In a retrospective cohort study of patients with acute kidney injury and HUA, Martens et al. found that rasburicase more effectively lowered UA than allopurinol [22]. Between 2009 and 2015, in a hospital setting, 89 patients were treated with rasburicase, either alone or in combination with allopurinol, while 61 patients received only allopurinol. Evidence related to the clinical benefit of rasburicase in renal recovery was inconclusive, supporting the need for additional comparative studies of rasburicase vs. allopurinol. These distortions may occur because of differences in factors other than those being studied (confounders). Observed or unobserved confounder effects of randomized controlled trials (RCTs) are balanced by random assignment to each Tx group. Propensity score matching (PSM) used in the Cairo and Martens studies was intended to control for observed confounders, and PSM also was utilized in this study.

The objective of this study was to determine whether a significant difference exists in the proportion of mortalities that the treating physician, in the RW setting, indicated were the direct result of TLS following Tx with allopurinol monotherapy or rasburicase monotherapy among those patients who may or may not have subsequently developed TLS after Tx with anti-HU agent. The null hypothesis before matching was that no significant difference exists between patients receiving allopurinol only and patients receiving rasburicase only regarding whether they died as a direct result of TLS after the start of anti-HU Tx. This is the first US, RW, PSM, confounder-controlled, observational mortality comparison study of patients treated with rasburicase vs. allopurinol monotherapy who had hematological malignancies and were at risk of HUA of TLS.

## Methods

The data of the study were drawn from a RW United States (US)-representative, physician-based and blinded, retrospective observational database of patients who were treated during the previous 12 months for being at risk of hyperuricemia/TLS potential and who had one of seven included hematological cancers, fielded June–September 2021. To obtain a nationally representative sample of study-qualified patients, the authors selected from a random, geographically representative sample of US medical oncologists and hematologists treating pediatric or adult patients from community practices, academic and non-academic hospitals, and outpatient clinics in the US who had treated at least one study-qualified patient during the previous 12 months. These randomly selected physicians were invited electronically to participate in a study of patients with target hematological malignancies (acute myeloid leukemia, acute lymphoblastic leukemia, chronic myeloid leukemia, chronic lymphocytic leukemia, multiple myeloma, or non-Hodgkin’s lymphoma (Burkitt or diffuse large B-cell lymphoma)) who had received therapeutic regimens posing risk for HUA and had potential for TLS. Of the 323 physicians who visited the study website, 242 were randomly selected from national physician-specialty panels, and 81 were randomly selected from the corresponding specialties in the American Medical Association (AMA) Physician Masterfile. Each potential and study-qualified physician in the US had a known, non-zero probability of being selected for the study, an essential component of a representative physician sampling frame of US geographical representativeness. The distribution of physicians participating in the study by US census region (South (34%), Northeast (26%), Midwest (20%), and West (20%)) was statistically similar to the actual specialty distributions listed in the AMA Physician Masterfile.

To determine the probability of study selection for each patient for whom a physician provided information, the national patient incidence data were used for each tumor type from the Surveillance, Epidemiology, and End Results (SEER) program of the National Cancer Institute. A total of 266 of these 323 physicians participated by providing requested data; the study participation rate was 82.4%.

To randomize patient selection for the 2021 chart audit study, each participating physician was instructed to select up to the last four study-qualified patients seen within the previous 12 months. The physicians provided anonymized data for 715 randomized patients with hematological malignancy treated in the previous year for HUA risk and TLS potential.

This report focuses on a subset of these data: patients who received anti-HUA monotherapy with either rasburicase or allopurinol, who did not experience pre-HUA Tx TLS nor spontaneous TLS, and who could be matched to an appropriate degree on potential confounders for which study data were available.

**Fig. 1:**
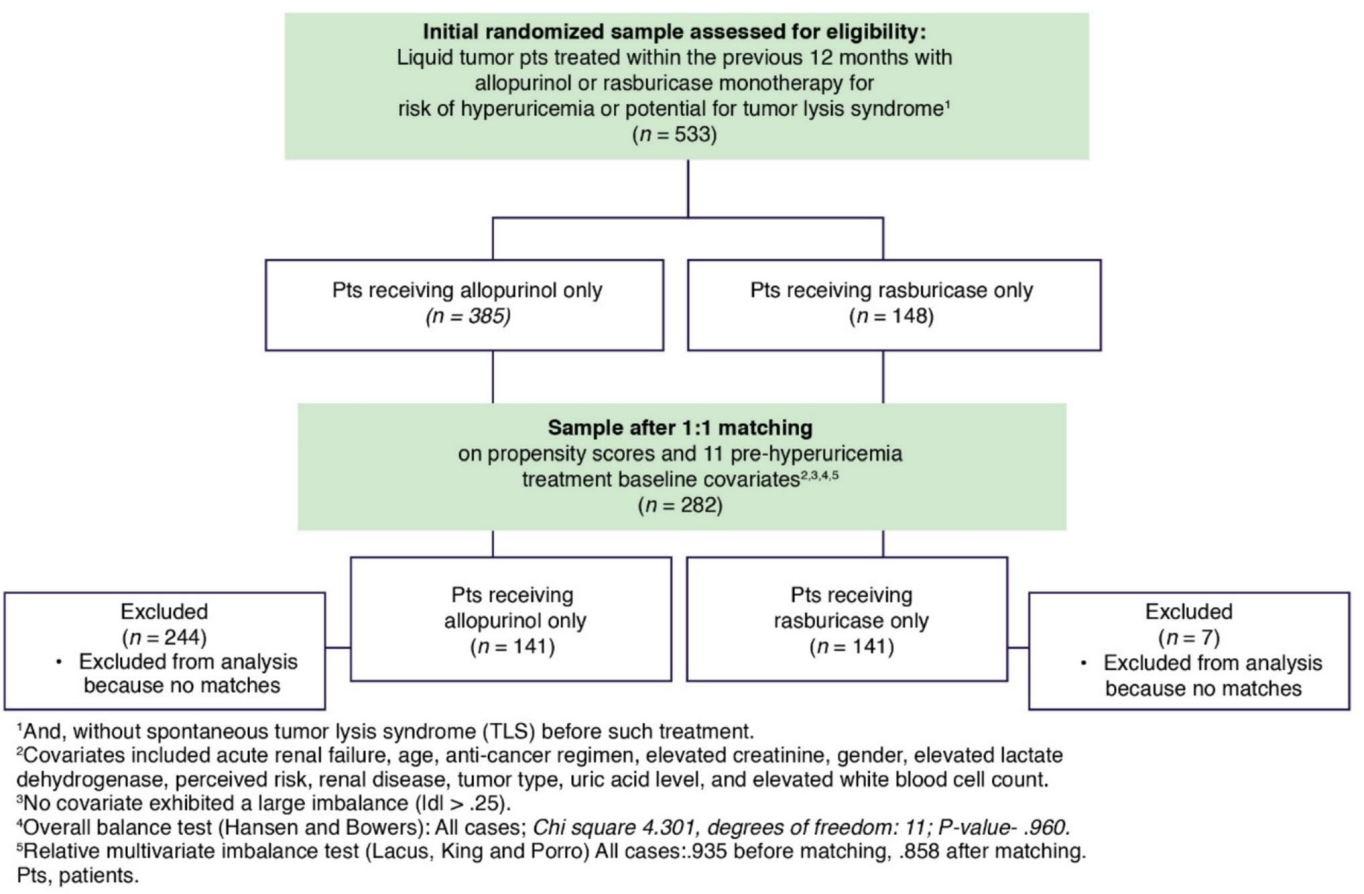
US representative, randomized sample of patients treated with rasburicase or allopurinol monotherapy within the previous 12 months

PSM was selected to control for observed confounders. This method to infer causal influences with observational data was employed because random assignment of subjects to Tx groups is not feasible as in a clinical trial. PSM incorporates the pairing of Tx groups (rasburicase and allopurinol in this study) with similar PSs (the probability of developing TLS) while balancing the effects of highly influential covariates, including the discarding of all unmatched units. IBM SPSS Statistics Essentials for R and MatchIt software were used to match patients treated with allopurinol only and patients treated with rasburicase only (ratio of 1:1) on TLS risk regardless of whether a patient later developed post-anti-HUA Tx TLS. The PSM analysis included only those patients from the 2021 observational study who received either rasburicase or allopurinol monotherapy and excluded patients with spontaneous TLS or TLS before anti-HUA Tx, leaving 533 potential subjects, 282 (141 in each Tx group) of whom were successfully nearest-neighbor matched in a ratio of 1:1 on PS and 11 predictive pre-anti-HUA Tx baseline covariates: acute renal failure, age, anti-cancer regimen, elevated creatinine, birth gender, elevated lactate dehydrogenase (LDH), perceived risk (low, intermediate, high), renal disease, tumor type, UA laboratory levels, and elevated white blood cell (WBC) count. These data were pulled from the original chart audit survey, which captured each patient’s demographics, co-morbidities, as well as anti-cancer, anti-hyperuricemia, and, if applicable, anti-TLS diagnosis and treatment histories. Of the 533 initial subjects, 7 rasburicase-treated patients and 244 allopurinol-treated patients could not be matched, and they were discarded (Figure 1). Via binary logistic regression analysis, we found each of the 11 covariates was highly predictive and appropriate for the analysis of physician-determined TLS-caused mortality among the 282 matched patients (correct prediction: 95.4%—chi-square: 35.214; *p* = 0.004) [13]. Data assessments of the 282 matched patients included mean UA levels, anti-cancer Tx (Tx), and, as available, timing of death relative to HUA Tx.

Caliper-based pair-matching was used on the probability of Tx selection of rasburicase- or allopurinol-treated patients using calipers of width equal to 0.2 of the standard deviation of the logit of the PS (d score). The Heinze and Jüni study supports this methodology that matching on the logit of the propensity score with a caliper width of 0.2 standard deviations of the logit of the PS may be superior to other methods used in the medical literature and is robust against misspecification of the propensity model [23].

## Results

Demographics of the 282 patients (*n* = 141 in each Tx group) are depicted in Table 1.

**Table 1:** Patient demographics and pre-HU treatment baseline factors

Variables	Allopurinol [A]* Treated *n* = 141		Rasburicase [B]* Treated *n* = 141			Survivors [C]* n = 269		Non-survivors [D]* n = 13		
US census regions	%	***n***	%	***n***	***p***-value	%	***n***	%	***n***	***p***-value
Northeast	23.4%	33	29.1%	41	0.28	24.5%	66	61.5% [C]	8	0.00
Midwest	20.6%	29	19.9%	28	0.90	20.4%	55	15.4%	2	0.66
South	36.2%	51	34.8%	49	0.79	36.1%	97	23.1%	3	0.34
West	19.9%	28	16.3%	23	0.43	19.0%	51	0.0%	0	0.08
Age (mean years, standard deviation, age range)	56.3, SD 19.1, 3–89	141	53.1, SD 18.2, 3–89	141	0.15	54.8, SD 18.7, 3–89	269	52.8, SD 18.5, 3–89	13	0.71
Gender										
Male	69.5%	98	70.2%	99	0.90	83.3% [D]	224	53.8%	7	0.01
Female	30.5%	43	29.8%	42		16.7%	45	46.2%	6	
Tumor type										
Acute myeloid leukemia	11.3%	16	13.5%	19	0.58	12.6%	34	7.7%	1	0.60
Acute lymphoblastic leukemia	13.5%	19	13.5%	19	1.00	11.9%	32	46.2% [C]	6	0.00
Chronic myeloid leukemia	17.0%	24	9.9%	14	0.08	14.1%	38	0.0%	0	0.15
Chronic lymphocytic leukemia	10.6%	15	15.6%	22	0.21	13.4%	36	7.7%	1	0.55
Multiple myeloma	21.3%	30	12.8%	18	0.06	16.4%	44	30.8%	4	0.18
Non-Hodgkin lymphoma (Burkitt & DLBCL)	26.2%	37	34.8%	49	0.12	31.6%	85	7.7%	1	0.07
Pre-HUA Tx HCP-perceived TLS risk level (high & intermediate)	86.5%	122	85.1%	120	0.74	85.9%	231	84.6%	11	0.90
Pre-HUA Tx UA (mg/dL)	12.2, SD 15.7	141	10.9, SD 12.8	141	0.45	11.4, SD 14.5	269	13.5, SD 11.2	13	0.61
Pre-HUA Tx clinical markers cited by HCPs										
Elevated creatinine**	29.8%	42	28.4%	40	0.80	30.1%	81	7.7%	1	0.08
Acute renal failure	12.8%	18	15.6%	22	0.50	13.0%	35	38.5% [C]	5	0.01
Renal disease	2.1%	3	2.8%	4	0.70	2.6%	7	0%	0	0.56
Elevated LDH**	33.3%	47	31.2%	44	0.71	33.5%	90	7.7%	1	0.05
Elevated WBC**	12.8%	18	13.5%	19	0.86	13%	35	15.4%	2	0.80
Dialysis during target study period	7.1%	10	7.1%	10	1.00	5.9%	16	30.8% [C]	4	0.00
Statistically significantly different anti-cancer Tx regimens										
Hyper-CVAD	1.4%	2	10.6% [A]	15	0.00	6.3%	17	0%	0	0.35
7 + 3 regimen	6.4% [B]	9	0.7%	1	0.00	3.7%	10	0%	0	0.48
RVd	7.8% [B]	11	2.1%	3	0.01	5.2%	14	0%	0	0.40
R-EPOCH	5.0% [B]	7	0.7%	1	0.01	3.0%	8	0%	0	0.53
77 other, statistically similar anti-cancer Tx regimens (*p*-values > 0.050)***	79.4%	112	85.8%	121	0.16	81.8%	220	100%	13	0.09

For each significant pair, the letter of the smaller category appears in the category with the larger number and reflects significance at the 95% confidence level. CAUTION—small n’s in some segments

HCPs were asked to cite pre-HUA Tx clinical markers for their patients in the 2021 retrospective, observational survey, and all HCPs responded for all patients. Those responses were employed as a proxy for specific lab levels because the PS matching program does not permit missing data, and creatinine, LDH, and WBC levels were not available for all patients. In the 2021 study, elevated UA was defined as > 7.5 mg/dL; creatinine > 1.2 mg/dL; LDH > 333 IU/L; WBC > 11,000/mm^3^

% of patients in statistically similar regimens—no significant differences in 77 out of 81 matched single agent and/or combination agent regimens. The regimens taken by the 13 patients who died are as follows: single agents: cisplatin *n* = 1, decitabine *n* = 1, ibrutinib *n* = 1, oxaliplatin *n* = 1, rituximab *n* = 1; combination Tx: doxorubicin, bleomycin, vinblastine, dacarbazine *n* = 5, cytarabine + idamycin *n* = 1, R-CHOP *n* = 1, and vincristine, daunorubicin, dexamethasone, PEG asparaginase, *n* = 1

*DLBCL* diffuse large B-cell lymphoma, *HCP* healthcare professional, *HUA* hyperuricemia, *LDH* lactate dehydrogenase, *SD* standard deviation, *TLS* tumor lysis syndrome, *UA* uric acid, *US* United States; *WBC* white blood cell, *Tx* treatment, *mg/dL* milligrams per deciliter, *IU/L* international units per liter, *mm*^*3*^ cubic millimeter, *Hyper-CVAD* cyclophosphamide, vincristine, doxorubicin, dexamethasone; *7 + 3*, cytarabine (also called Ara-C) + most often daunorubicin, *RVd (VRd)* lenalidomide, bortezomib, dexamethasone, *R-EPOCH* rituximab + etoposide, prednisone, vincristine, cyclophosphamide, doxorubicin

**Fig. 2:**
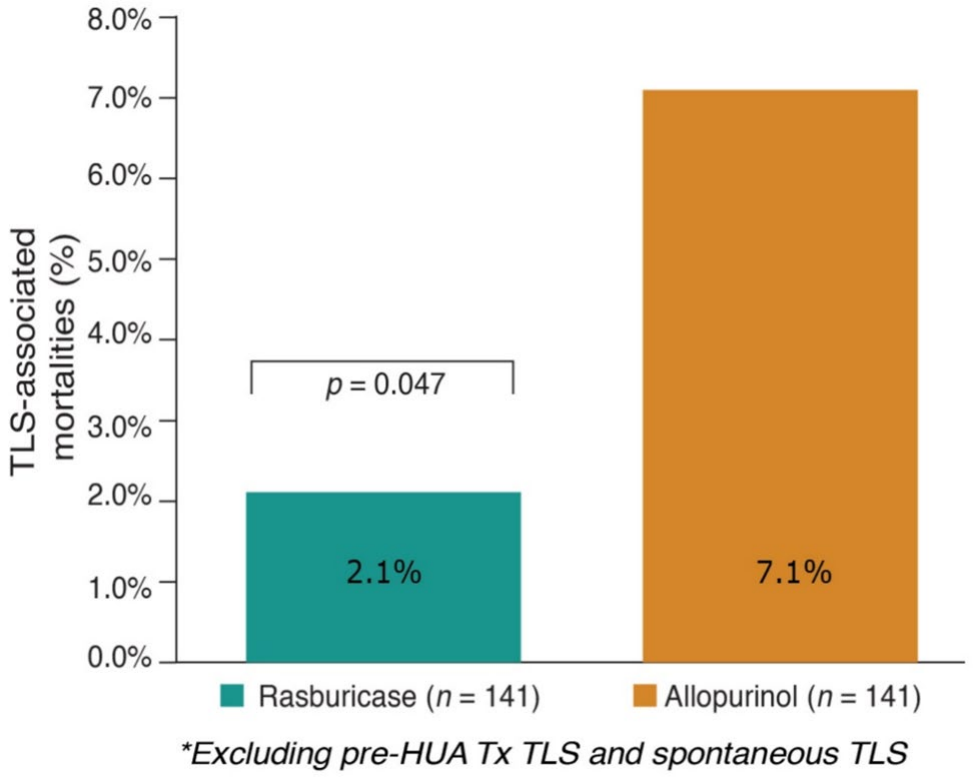
TLS-associated mortalities: post-HUA treatment in all matched patients (*n* = 282)*

There were no statistically significant demographic differences between the rasburicase and allopurinol Tx groups. On analyzing all 141 pairs of matched patients (*n* = 282), it was found that TLS-associated mortality was significantly less likely among rasburicase-treated patients (2.1% vs. 7.1%; *p* = 0.047) (Figure 2). Analyzing a 63-patient subset who developed TLS after the start HU treatment, TLS-associated fatalities were even less likely among rasburicase pts, 3 of 36 rasburicase vs. 10 of 27 allopurinol pts (*p*-value 0.005)). Study physicians stated in the chart audit survey instrument that all of the mortalities cited were in association with the complications of TLS.

**Fig. 3:**
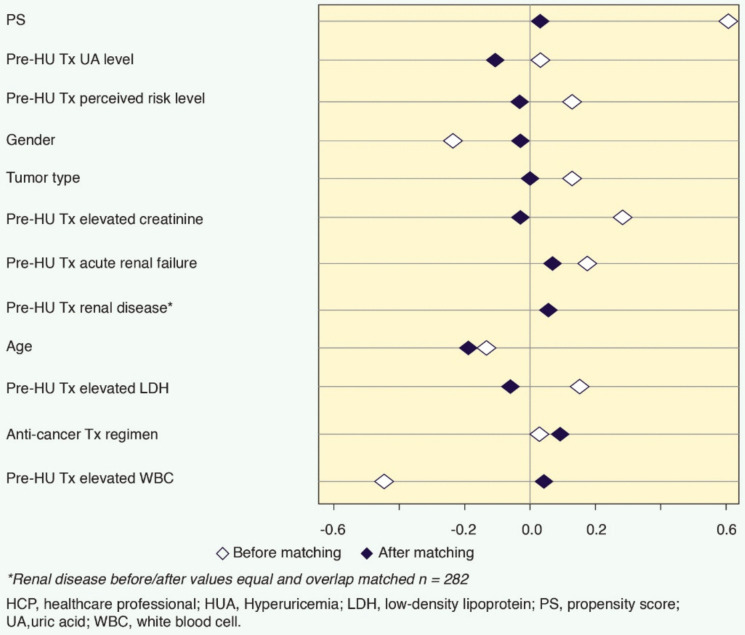
Pre- and post-matching of 11 covariates and PS

Figure 3 provides evidence of the extent to which the matching efforts corrected for overall covariate and individual baseline covariate imbalances before and after matching. The logit of the overall PS was about 0.6 before matching but reduced to near 0 post-matching. Each of the 11 baseline covariates after matching was < 0.2 of the standard deviation of the logit of the PS. Covariates requiring the greatest reduction were elevated WBC, creatinine, and LDH levels and birth gender.

No covariate exhibited a large imbalance (│*d*│>0.25), nor did the overall relative imbalance difference of the groups (0.077) before and after matching. The match of the two groups was indicated by the Hansen and Bowers balance test and the Iacus, King, and Porro relative multivariate imbalance before and after matching test (Figure 3) [24, 25].

**Fig. 4:**
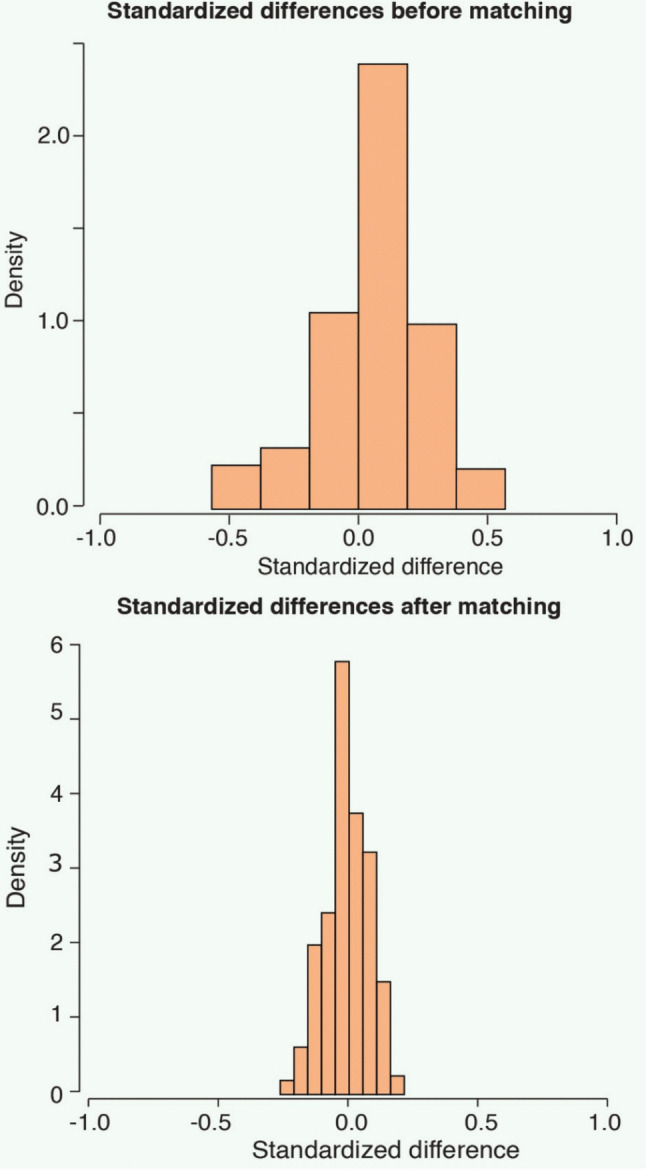
Pre- and post-matching density of standardized differences

There was a significant improvement in the density of overall standardized differences before and after matching. This indicated most of the standardized differences between the two groups were clustered near 0 (Figure 4).

**Fig. 5:**
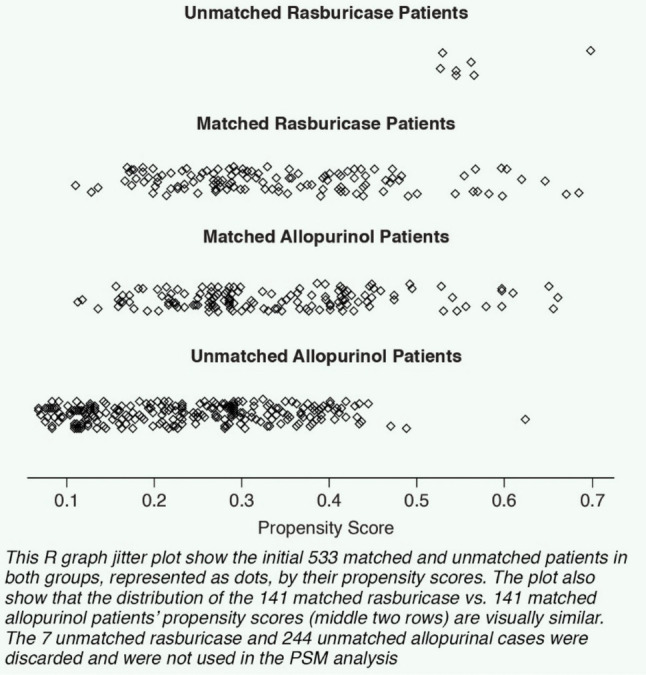
Distribution of propensity scores

Similar evidence of match is provided by a jitter plot in Figure 5, showing the distribution of matched rasburicase vs. allopurinol scores (middle two rows) as visually similar.

**Fig. 6:**
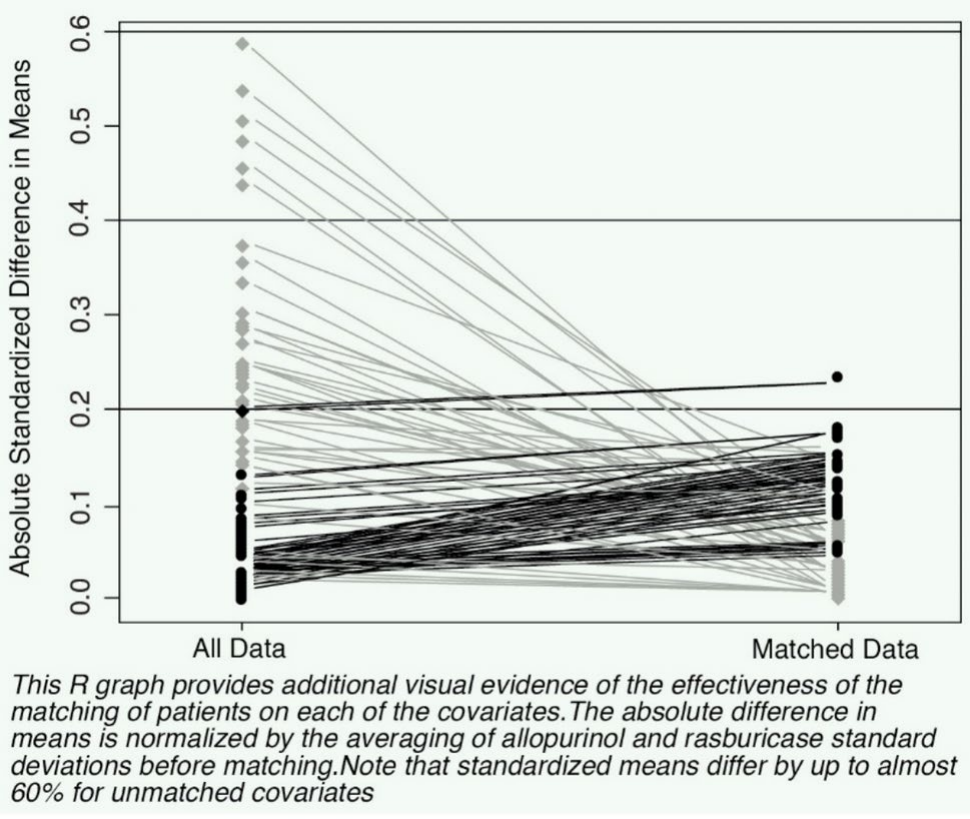
Standardized differences minimized

Figure 6 shows that the absolute difference in means was normalized by the averaging of allopurinol and rasburicase standard deviations before matching. The standardized means differ by up to almost 60% for unmatched covariates.

Comparing the 13 patients who died with 269 patients who did not, no significant difference in mean UA (13.5 mg/dL vs. 11.4 mg/dL, respectively) was found. Rituximab, cyclophosphamide, doxorubicin, vincristine, and prednisone regimen (R-CHOP) was the predominant Tx among those who did not die (15%, *n* = 41). Only 1 (7.7%) of 13 patients who died received R-CHOP. Dates of death were not reported in the 2021 anonymized chart study, but there were sufficient data points regarding HUA Tx duration as well as the number of days or weeks between Tx and the physician’s entry of the patient cases in the survey to determine that 7 patients died within 2 weeks or less of allopurinol (*n* = 6) or rasburicase (*n* = 1) monotherapy (timing could not be established for the other 6).

## Discussion

This study is the first reported US representative study comparing rasburicase and allopurinol on risk of mortality. In this regression analysis, including PSM on 11 pre-anti-HUA-Tx, mortality was found to be significantly lower after monotherapy with rasburicase vs. allopurinol. Previous comparison trials of rasburicase vs. allopurinol in children (Goldman et al.) and in adults (Cortes et al.) were not powered to determine significant differences in survival rate or mortality [1, 26]. Goldman et al. demonstrated a significant reduction in the UA exposure over 96 h (AUC0-96) between rasburicase and allopurinol in children with TLS or at risk of TLS (128 ± 70 mg/dL/h vs. 329 ± 129 mg/dL/h: *p* <0.0001) [1] and demonstrated an 86% vs. 12% reduction in plasma UA levels at 4 h after rasburicase vs. allopurinol, respectively (*p* <0.0001) [1]. Cortes et al. demonstrated a normalization of UA levels (≤7.5 mg/dL) in 87% of adult patients with hematological malignancies with TLS or at risk of TLS following rasburicase monotherapy and only 66% following allopurinol monotherapy (*p* < 0.0001). In patients with HUA at study entry, 90% of patients receiving rasburicase monotherapy vs. 53% of patients receiving allopurinol monotherapy had normalization of UA (*p* < 0.015) [26]. Goldman et al. reported that the UA AUC of 144 mg/dL/h was significantly reduced when treated with rasburicase compared to allopurinol monotherapy, with values recorded at 77 ± 57 mg/dL/h vs. 646 vs. 285 mg/dL/h, respectively (*p* < 0.0001) [1]. This suggests that rapidity to normalization of UA levels and reduced exposure to higher UA levels may significantly decrease the risk of mortality associated with TLS in patients with hematological malignancies. Hypothetically, this may be secondary to improvement of renal function and/or prevention of acute kidney injury following rasburicase vs. allopurinol monotherapy. Galardy et al. demonstrated a significant improvement in estimated glomerular filtration rate following rasburicase monotherapy in children with advanced-stage B-cell lymphoma undergoing induction chemotherapy (day 1: glomerular filtration rate 55 mL/min/1.73 m^2^ vs. day 7: 136 mL/min/1.73 m^2^: *p* < 0.0007) [27].

Cairo et al. have also demonstrated a significant increase in the hospitalization length of stay, number of intensive care unit days, and amount of total hospital cost for adults with hematological malignancies with TLS or at risk of TLS with acute kidney injury, presumably related to TLS [21]. Adult patients with hematological malignancies with TLS or at risk of TLS treated with rasburicase vs. allopurinol had a mean reduction of total hospitalization by 5 days (*p* < 0.02), a mean reduction of intensive care unit stay by 2.5 days (*p* < 0.001), and a significant reduction in costs ($20 038) (*p* < 0.02) [21].

The findings of this comparative mortality study of rasburicase monotherapy vs. allopurinol monotherapy are based on a scientifically sound, RW observational methodology, the only type of study design that makes it possible to obtain a nationally representative probability sample from which study findings can be generalized. A major challenge in this study was to minimize the effects of confounders, a complication in observational studies [28]. PSM was used to minimize, equilibrate, and balance the two Tx groups on study-measured confounding factors and to reduce selection bias. The post-matching analyses indicate that these efforts were successful. This study is not without limitations. PSM was used to equilibrate confounding variables to control for observed baseline factors. The authors know that PSM cannot assess and balance all the relevant factors that may influence the clinical management of patients, including comparable quality of care or physicians’ Tx choices based on their assessments of patient risk in a retrospective, observational RW study. It is not known the extent to which the study covariates are comprehensive. The matching algorithms did not permit use of any covariates with missing data. Limiting this study to only patients receiving monotherapy to isolate individual therapy Tx effects is another limitation because some patients in the RW setting are simultaneously treated with allopurinol and rasburicase. Conducting a prospective randomized trial in patients with hematological malignancies with HUA who are at risk of TLS comparing rasburicase vs. allopurinol with a primary endpoint of mortality would be ethically challenging: Unless contraindicated, rasburicase is the Tx of choice in patients with hematological malignancies with TLS or high risk of developing TLS and in intermediate-risk patients with certain clinical markers, while allopurinol is one of the Txs recommended in low- and intermediate-risk patients [29]. It was determined that 95% of the matched patients had no contraindications to any “other therapies.”

## Conclusion

Study results indicate (1) rasburicase when compared with allopurinol significantly reduces TLS-associated mortality, and (2) PS matching successfully corrects before and after overall covariate and individual baseline covariate imbalances in comparing mortality. It is well known that rasburicase and allopurinol control uric acid levels via distinctly different mechanisms; rasburicase enzymatically converts existing uric acid to allantoin while allopurinol works via inhibiting the production of uric acid. Future studies will be required to determine the exact mechanisms responsible for these observations. Unless contraindicated, rasburicase is the Tx of choice in patients with hematological malignancies with TLS or at high risk of developing TLS.

## Data Availability

No datasets were generated or analysed during the current study.

## Declarations

### Previous presentations

This material was presented in part by the authors as posters, including at the Annual Meeting of the Society of Hematologic Oncology, September 2022, Houston, TX, USA; at the Annual Meeting and Exposition of the American Society of Hematology, December 2022, New Orleans, LA, USA; and at the European Society for Medical Oncology (ESMO), Madrid, Spain, October 20–24, 2023.
